# Cloud Model Bat Algorithm

**DOI:** 10.1155/2014/237102

**Published:** 2014-05-19

**Authors:** Yongquan Zhou, Jian Xie, Liangliang Li, Mingzhi Ma

**Affiliations:** College of Information Science and Engineering, Guangxi University for Nationalities, Nanning, Guangxi 530006, China

## Abstract

Bat algorithm (BA) is a novel stochastic global optimization algorithm. Cloud model is an effective tool in transforming between qualitative concepts and their quantitative representation. Based on the bat echolocation mechanism and excellent characteristics of cloud model on uncertainty knowledge representation, a new cloud model bat algorithm (CBA) is proposed. This paper focuses on remodeling echolocation model based on living and preying characteristics of bats, utilizing the transformation theory of cloud model to depict the qualitative concept: “bats approach their prey.” Furthermore, Lévy flight mode and population information communication mechanism of bats are introduced to balance the advantage between exploration and exploitation. The simulation results show that the cloud model bat algorithm has good performance on functions optimization.

## 1. Introduction

Metaheuristics is a new method for stochastic optimization; in recent years, more and more different metaheuristic algorithms have been proposed, such as particle swarm optimization (PSO) [1], differential evolution (DE) [2], and bat algorithm (BA) [3], and some novel metaheuristic algorithms are proposed. The bat algorithm was proposed by Xin-She Yang in 2010, which is inspired by the echolocation behaviour of microbats. The bat algorithm controls the size and orientation of bats moving speed through adjusting the frequency of each bat and then moves to a new location; the intensive local search is controlled by the loudness and pulse emission rate. To some extent, PSO is a special case of suitably simplified BA. Due to the fact that BA combines with the advantages of swarm intelligence, which utilizes a balanced combination of the advantages of the standard PSO and the intensive local search controlled by the loudness and pulse rate, BA is widely researched in different field applications. BA has some advantages over other algorithms, and the number of adjustable parameters is fewer. Consequently, BA has been used for solving engineering design optimization [4–6], classifications [7], fuzzy cluster [8], prediction [9], neural networks, and other applications.

The cloud model is proposed by Li et al. in 1995, which is a model of the uncertain transition between a linguistic term of qualitative concept and its numerical representation [10]. In recent years, the cloud model is applied in the field of metaheuristics, such as cloud model based genetic algorithm (CGA) [11] and cloud model based evolutionary algorithm (CBEA) [12, 13]. In this paper, the bat algorithm was used for reference, the echolocation mechanism based on cloud model was remodeled, and two mechanisms were introduced: population information communicating of each individual and random Lévy flight; a cloud model bat algorithm (CBA) was proposed, and the purpose is to improve the convergence rate and precision of bat algorithm. At the end of this paper, combination strategies and parameter settings of CBA are discussed, several appropriate parameters are selected, and eight typical benchmark functions are tested, and the test results show that the proposed algorithm is feasible and effective.

## 2. Behaviors of Bats and Cloud Model

### 2.1. Flight and Echolocation of Bats

Bats are the only volitant mammals in the world; after tens of millions of years of evolution, there are nearly 1,000 species of bats. Bats have powered flight ability, which is much more complex than glide; their flight can generate complex aerodynamic tracks, and the flight is accompanied with local self-similarity [14]. Many microbats have amazing echolocation; these bats can emit a very loud and short sound pulse and receive the echo that reflects back from the surrounding objects by their extraordinary big auricle. Then, they analyze this feedback information of echo in their subtle brain. They not only can discriminate direction for their own flight pathway according to the echo but also can distinguish different insects and obstacles, to hunt prey and avoid collision effectively in the day or night. Bats minimize the conspicuousness of their echolocation call to potential insect prey by reducing call intensity and by changing the frequencies in the call [15]. Furthermore, the echolocation signal that one individual bat uses to collect information can simultaneously serve as a communication function, allowing, for example, group members to remain in contact with one another. Echolocation call plays a crucial and hitherto underestimated role for social communication in a highly mobile and gregarious nocturnal mammal and thus facilitates social communication in bats population [16].

### 2.2. Bat Algorithm

In simulations, they use virtual bats naturally, to define the updated rules of their positions *x*_*i*_ and velocities *v*_*i*_ in a *D*-dimensional search space. The new solutions *x*_*i*_^*t*^ and velocities *v*_*i*_^*t*^ at time step *t* are given by

(1) $$\begin{array}{c} f_{i}=f_{\min}+\left(f_{\max}-f_{\min}\right)\beta ,\\ v_{i}^{t}=v_{i}^{t-1}+\left(x_{i}^{t}-x_{*}\right)f_{i},\\ x_{i}^{t}=x_{i}^{t-1}+v_{i}^{t}, \end{array}$$

where *β* ∈ [0,1] is a random vector drawn from a uniform distribution. Here, *x*_∗_ is the current global best location (solution) which is located after comparing all the solutions among all the *n* bats.

For the local search part, once a solution is selected among the current best solutions, a new solution for each bat is generated locally using random walk:

(2) $$\begin{array}{c} x_{\text{new}}=x_{\text{old}}+\varepsilon A_{t}, \end{array}$$

where *ε* ∈ [−1,1] is a random number, while *A*_*t*_ = 〈*A*_*i*_^*t*^〉 is the average loudness of all the bats at this time step.

Furthermore, the loudness *A*_*i*_ and the rate *r*_*i*_ of pulse emission have to be updated accordingly as the iterations proceed. These formulas are

(3) $$\begin{array}{c} A_{i}^{t+1}=\alpha A_{i}^{t},\\ r_{i}^{t+1}=r_{i}^{0}\left[1-\exp\left(-\gamma t\right)\right], \end{array}$$

where *α* and *γ* are constants.

Based on these approximations and idealization, the basic steps of the bat algorithm [3] can be summarized as the pseudocode shown in Algorithm 1.

### 2.3. Lévy Flight

**Figure 1:**
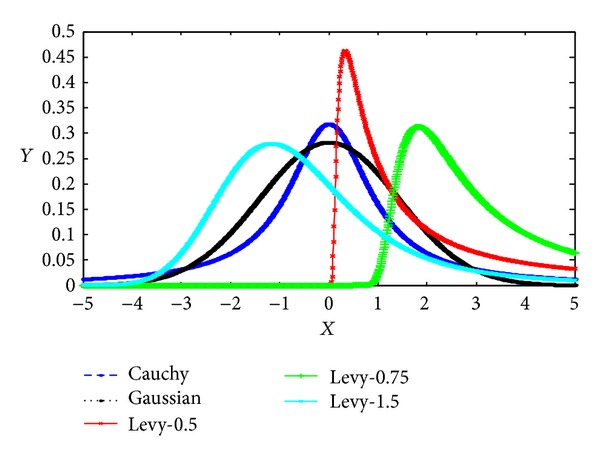
The PDF curve of the three distributions.

Lévy flight is a random walk in which the step-lengths have a probability distribution that is heavy-tailed. Lévy flight has several properties: “heavy tails,” statistical self-similarity, random fractal characteristics, and infinite variance with an infinite mean value [17]. Lévy distribution, Gaussian distribution, and Cauchy distribution, which is a  *α*  stable distribution; however, probability density function (PDF) curves of Gaussian distribution and the Cauchy distribution are symmetrical; Lévy distribution is not symmetrical [18]. Probability density function of Lévy distribution on *x* > *μ* is

(4) $$\begin{array}{c} \text{Lévy}\sim f\left(x\right)=\sqrt{\frac{C}{2\pi }}\frac{e^{-C/2(x-\mu )}}{{\left(x-\mu \right)}^{3/2}}, \end{array}$$

where *μ* is the location parameter and *C* is the scale parameter. PDF curve of the three distributions is presented in Figure 1.

Studies have shown that flight behaviour of many animals and insects has demonstrated the typical characteristics of Lévy lights. A recent study by Reynolds and Frye shows that fruit flies explore their landscape using a series of straight flight paths punctuated by a sudden 90° turn, leading to a Lévy flight-style intermittent scale free search pattern [19]. Studies on human behaviour such as the Ju/'hoansi hunter-gatherer foraging patterns also show the typical feature of Lévy flights [20]. Subsequently, due to the remarkable properties of stable Lévy distribution, Lévy flight has been applied to optimization and optimal search [21], and preliminary results show its promising capability.

### 2.4. Cloud Model

Cloud model build a transformational bridge between a linguistic term of qualitative concept and quantitative representation, which reflects randomness, fuzziness, and the relationship between randomness and fuzziness of uncertainty in knowledge representation [22, 23]. The cloud and cloud droplets are defined as follows.

Let *U* be the set *U* = {*x*}, as the universe of discourse, and let *C* be a linguistic term associated with *U*. The membership degree of *x* in *U* to the linguistic term *C*, *μ*(*x*), is a random number with a stable tendency. *μ*(*x*) takes the values in [0, 1]. A membership cloud, or compatibility cloud, is a mapping from the universe of discourse *U* to the unit interval [0, 1]. That is,

(5) μ(x):U⟶[0,1],∀x∈U, x⟶μ(x).

The distribution of *x* in universe of discourse *U* is called cloud and each *x* is called a drop of cloud [22].

A normal cloud is defined with three digital characteristics, expected value *Ex*, entropy *En*, and hyper entropy *He* and a cloud, namely, *C*(*Ex*, *En*, *He*). Expectation *Ex* is the position at *U* corresponding to the center of gravity of the cloud. In other words, the element *Ex* in the universe of discourse is fully compatible with the linguistic term. The entropy *En* is a measure of the coverage of the concept within the universe of discourse. In other words, *En* is defined by the bandwidth of the mathematical expected curve (MEC) of the normal cloud showing how many elements in the universe of discourse could be accepted to the linguistic term, the greater *En*, and the broader coverage. It can be also considered as a measure of fuzziness of the concept, representing the scope of the universe of discourse that can be accepted by the concept. The hyper entropy *He* is the entropy of the entropy *En*. It is a measure of dispersion of the cloud drops; it can be used a measure of thickness of the cloud, which not only reflects the randomness of samples appearing that represent qualitative concepts value but also reveals the relatedness between fuzziness and randomness.

**Figure 2:**
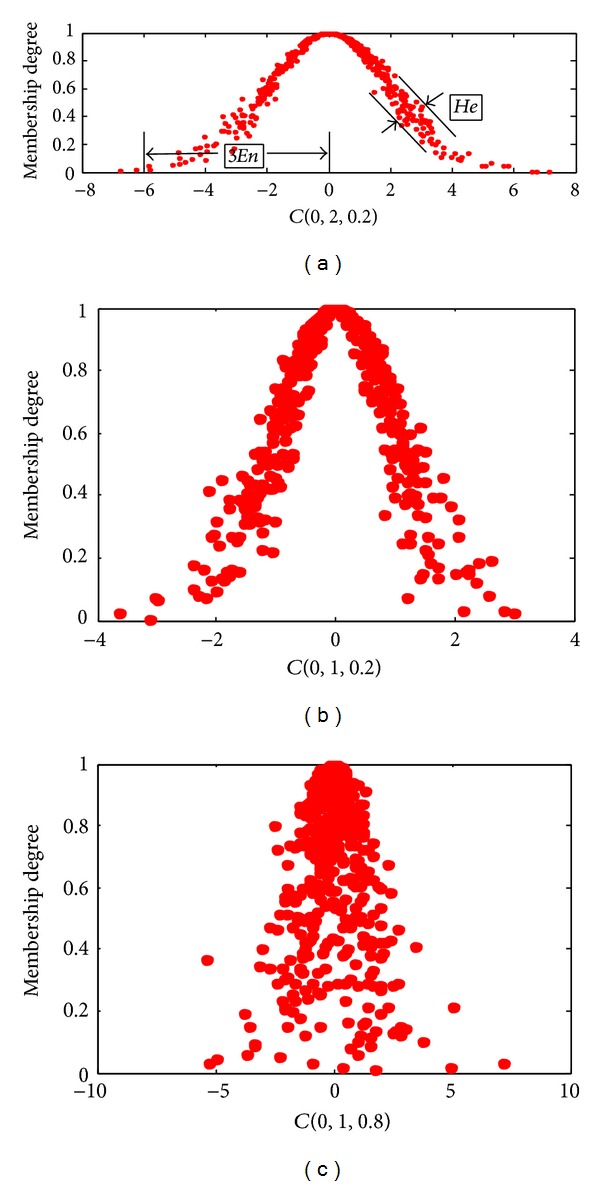
The cloud with different digital characteristics. *C*(0,2, 0.2) denote a cloud, 0 is expectation, 2 is entropy, and 0.2 is hyper entropy.

Normal cloud model makes full use of the universality of the normal distribution and normal membership function, which not only broaden the formation conditions of the normal distribution but also make the normal membership function be the expectation of the random membership degree; the randomness and fuzziness are represented uniformly by entropy and then the theoretical basis of universality of the normal cloud model is established [24]. Cloud model has the 3*σ* characteristics; there are 99.7% drops of cloud located in [*Ex* − 3*En*, *Ex* + 3*En*]. These drops of cloud are generated by the normal cloud generator. Atomized feature of the cloud model: the drops of cloud spread around while the hyper entropy is increasing, but many drops still stand in the central area of the cloud, which can be used to adjust the strategies of the evolution and help to escaping from local optima [11]. The clouds with different digital characteristics are depicted in Figure 2.

## 3. Cloud Model Bat Algorithm

Bats prey by emitting pulse with a certain frequency and detection of the echo; they communicate with each other using echolocation call. This paper assimilates its principle to idealize some of the echolocation characteristics of microbats. Based on the excellent characteristics of cloud model on uncertainty knowledge representation, a bat algorithm based on cloud model was proposed (cloud model bat algorithm, CBA).

### 3.1. Knowledge Representation of Bat Cloud

In order to depict the CBA, the habits of bats are used for reference, taking advantage of the excellent properties of cloud model. Firstly, representation of relevant knowledge needs to be described; several concepts certain about CBA were given as follows.

(*1) Optimizing Generation*. Optimizing generation indicates the number of iteration circles in the algorithm; each iteration circle may include several times replacement of population, simply, namely, *t*.

*(2) Individual*. In CBA, each bat is treated as an individual; when it is in flight, the position of each bat *x*_*i*_^*t*^ signifies a candidate solution of optimization problem, where *i* is the number of individuals and *t* is the optimizing generation. For the high-dimensional optimization, *x*_*i*_^*t*^ represent a vector under high-dimensional space, correspondingly, where each dimension denotes an attribute of the solution of optimization problem.

*(3) Fitness Function*. Fitness function denotes adaptation degree of each individual aiming at their located environment in the community. It is used to evaluate the individual and decides which individual to retain or eliminate. Fitness function usually is the expression of costs, profitability, variance, and so on.

*(4) Population Bats Cloud*. The bats live and prey together, and many bats constitute a community. A cloud was generated, which is to represent the distribution characteristics of the same dimension of all individuals, called population bats cloud, namely, *Pbc*_*j*_(*Ex*, *En*, *He*), where *j* represents the *j*th dimension of population and *Ex*, *En*, and *He* are three digital characteristics of cloud model.

*(5) Individual Experience*. It denotes these individuals that are able to remember their own history during the process of optimization. In the proposed algorithm, bats can memorize their own best location *x*_*p*best_ during moving. Its main purpose is to guide the flight of bat and to promote the communication among the population.

*(6) Population Elite*. In this proposed algorithm, population elite denotes the position of the optimal individual, namely, *x*_*g*best_; the population elite *x*_*g*best_ will be saved and be used in swarm information communication.

### 3.2. Cloud Model Bat Search Algorithm

In this paper, at the basis of original BA and the habits of bats, based on the cloud model and Lévy flights, cloud model bat algorithm is proposed under idealized simulation of echolocation of bats. For simplicity, some idealized rulesareas follows.

1. Using the echolocation, bats not only can identify the direction, measure the distance, and determine the current status of their prey but also can avoid collision, distinguish obstacles, and prey from background clutter. This paper only simulates that bats search for a prey using echolocation mechanism in a search space under ideal environment, where the position of prey means an optimal solution of the problem; each position of bats indicates a candidate solution of optimization problem. Bats may not prey their target, but they gradually approach the target, close to the prey, approximately regard as successful preying under a certain tolerance.
2. Each bat flies randomly with frequency *f*_*i*_^*t*^; the position *x*_*i*_^*t*^ moves under the adjustment of frequency *f*_*i*_^*t*^. The frequency *f*_*i*_^*t*^ resembles an adjustment coefficient of step length, and frequency *f*_*i*_^*t*^ of sound pulse is changeable, where *t* is the optimizing generation.
3. The adjustment of frequency *f* caused the change of the wavelength *λ* (this is because of the fact that *λf* = *v* is a constant and *v* is the speed of sound in air; typically, *v* = 340 m/s); such wavelengths *λ* are in the same order of their prey sizes and help to locate the target. Generally, the frequency *f* is in a range [*f*_min⁡_, *f*_max⁡_], and each individual can communicate information with others by echolocation call in a population.
4. Each bat emits sonic pulse with emission rate *R*_*i*_ ∈ [0,1] and loudness *Ld*_*i*_. At the beginning of prey, bats have a smaller *R*_*i*_ and larger *Ld*_*i*_. During the process of locating prey, the pulse emission rate increases and loudness reduces once the bat searches for target traces, which is figuratively indicated by “bat is approaching the target.”
5. In exploration of bats for prey, their flight features are accompanied by typical Lévy flight characteristic; many insects and animals have it as well. Exploration and traces its of to detect potential prey traces of a random flight.

On the basis of the above mentioned idealized rules, the properties of the cloud model are utilized which represents the membership degree of qualitative concept and reveals the relationship between randomness and fuzziness in uncertainty knowledge representation. According to normal cloud model generator with expectations, entropy, and hyper entropy, many drops of the cloud with quantitative transformation value corresponding to qualitative concept are produced. In this paper, updating the position of bats by cloud model, swarm information communication in each individual and random Lévy flights are introduced, a cloud model bat algorithm is proposed, and the steps of CMBA algorithm can be summarized as follows: 

*(1) Initialization.* Randomly initialize the position of each bat in the population and relevant parameters. 

*(2) Initial Evaluation.* Evaluate these initial positions using fitness function, and find out a population elite *x*_*g*best_. 

*(3) Bats Cloud Updating.* Generate bats cloud based on cloud model, and update the position of bats. 

*(4) Swarm Information Communication.* Information communication of bat population adopts a differential operator that is similar to mutation strategy “DE/best/2” in differential algorithm. 

*(5) Bats Random Lévy Flight. *Each bat randomly flights using Lévy flight. 

*(6) Population Evaluation. *For each population in steps (3)–(5), evaluate each individual by fitness function and find out and update the individual experience *x*_*p*best_ for each bat and population elite *x*_*g*best_ in each step. 

*(7) Pulse Emission Rate and Loudness Update*. The rate of pulse emission *R*_*i*_ and loudness *Ld*_*i*_ for each bat need to update when the achieved optimal solution after steps (3)–(5) is better than the optimal solution of last generation. 

*(8) Termination Judgment. t* = *t* + 1; execute steps (3)–(7) until *t* reaches a predefined maximum number of optimizing generation.

In this algorithm framework, three problems need to be solved: first of all, the formation of bat cloud model, second information communication of bat population, and third the updating of *R*, *Ld*.

#### 3.2.1. Formation of Population Bats Cloud Model

This paper simulates the moving of bats when several bats pursue and capture prey. Each bat expects to move toward the direction of prey (the optimal solution). In the search space, the entire population is trying to approximate the optimal solution; the position *x*_*i*_^*t*^ of each individual should move toward the optimum position. Consequently, the same dimensions of population have stable tendency. However, each individual has their own feature, and the implementations of position updating are random for each individual. Therefore, the characteristics of approximated process that bats have to approximate prey are simulated by cloud model. Sequentially, they adapt bat cloud model to depict the qualitative concept: “bats approach their prey.”

Normal cloud model of bat approach process utilizes the characteristics of cloud model that are the uncertain transition between qualitative and quantitative. The populations bats cloud *Pbc*_*j*_(*Ex*, *En*, *He*) analogize to cloud *C*(*Ex*, *En*, *He*), where the expected value *Ex* is the *j*th dimension of population elite *x*_*g*best_, the entropy *En* is the average loudness of all bats, and the hyper entropy *He* is the average pulse emission rate of all bats. The population bats cloud *Pbc*_*j*_(*Ex*, *En*, *He*) is a 1-dimensional normal cloud. In order to update the position of each individual, each dimension of new individual is generated by randomly selecting several drops of the cloud from cloud cluster, and then calculate the result which is mean of the membership degree of each selected drop multiplied by expected value *Ex*. The membership degree of each drop is the certainty degree of approximation expectation *Ex*. The computational formula is described as follows:

(6) $$\begin{array}{c} x_{ii}^{t+1}=\text{AVG}\left(\sum Ex\times RS\left(pbc_{i}\left(Ex,En,He\right)\right)\right), \end{array}$$

where *i* denotes *i*th individual, *j* denotes *j*th dimension, *Ex* = *x*_*g*best,*j*_, *En* = AVG(∑Rate_*i*_), *He* = AVG(∑Loudness_*i*_),  *RS*(·)  denotes a function of the randomly selected several records, and AVG(·)  denotes averaging function.

The pulse emission rate *R*_*i*_ increases and loudness *Ld*_*i*_ decreases while the iteration is increasing, and the entropy *En* and hyper entropy *He* therewith update. Consequently, different cloud clusters are generated, so those individuals gradually approach the target. Position of the bat is updated by population bats cloud *Pbc*_*j*_(*Ex*, *En*, *He*), which quantificationally represents the qualitative concept that bat approaches target. Sequentially, which reflect the determine tendency of swarm optimizing, meanwhile show the fuzziness and randomness of uncertainty knowledge representation.

The proposed algorithm introduces information communication in bat population under idealized conditions, which assure that the entire bat colony gets helpful information by communicating experience among individuals in a bat population. This mechanism guides these bats that are approaching prey fast.

Bats can emit sound pulses with certain frequency range. Generally, the frequency *f* is in a range [*f*_min⁡_, *f*_max⁡_] and the typical range is [0, 1] in implementation. This paper defines the frequency *f*  updating formula as follows:

(7) $$\begin{array}{c} f_{1i}^{t}=\left(\left(f_{1,\min}-f_{1,\max}\right)\frac{t}{n_{t}}+f_{1,\max}\right)\beta_{1}, \end{array}$$

(8) $$\begin{array}{c} f_{2i}^{t}=\left(\left(f_{2,\max}-f_{2,\min}\right)\frac{t}{n_{t}}+f_{2,\min}\right)\beta_{2}, \end{array}$$

where *β*_1_, *β*_2_ ∈ [0,1] is a random vector drawn from a uniform distribution, *f*_1,max⁡_ = *f*_2,max⁡_ = *f*_max⁡_, *f*_1,min⁡_ = *f*_2,min⁡_ = *f*_min⁡_, and *n*_*t*_ is a constant. The frequency *f* would be analogous to an adjustable parameter. The step length of individual moving is adjusted by adjusting frequency *f*. Meanwhile, it can be interpreted as bats adjusting their own position by adjusting their own frequency and communicating with other bats. Information communication of bat population adopts a differential operator that is similar to mutation strategy “DE/best/2” in differential algorithm, which is described as follows:

(9) $$\begin{array}{c} x_{i}^{t+1}=x_{g\text{best}}^{t}+f_{1i}^{t}\left(x_{r1}^{t}-x_{r2}^{t}\right)+f_{2i}^{t}\left(x_{r3}^{t}-x_{r4}^{t}\right), \end{array}$$

where *x*_*g*best_^*t*^ represents the current population elite after updating by bat cloud updating and *x*_*ri*_^*t*^ is *i*th individual randomly selected in the population after bat cloud updating.

In addition, the above mentioned mechanism accelerates the convergence rate, while the Lévy flight behavior is introduced to greatly ensure the swarm diversity against the premature convergence. Random Lévy flights are manipulated at the basis of individual experience *x*_*p*best_^*t*^, where *x*_*p*best_^*t*^ represents the current individual experience after swarm information communication. The random Lévy flights are used to improve the individual ability to escape from the local optima; simultaneously, this mechanism also assures the intensification. The detailed description is as follows:

(10) $$\begin{array}{c} x_{i}^{t+1}=x_{p\text{best}}^{t}+\mu \times \mathrm{sign}\left[\text{rand}-0.5\right]⊕\text{L}\overset{´}{\text{e}}\text{vy}, \end{array}$$

where *μ* is a random parameter drawn from a uniform distribution, sign ⊕ means entry-wise multiplications, rand ∈ [0,1], and random step length Lévy obeys Lévy distribution.

#### 3.2.2. Method of Pulse Emission Rate and Loudness Updating

The pulse emission rate *R*_*i*_ and loudness *Ld*_*i*_ of each bat will be adjusted suitably when it moves to a better position than last generation *t* − 1. In this paper, the updating formulas adopt (11). It is worth noting that the loudness and emission rates will be updated only if the final population elite *x*_*g*best_^*t*^ in current generation are better than the final population elite *x*_*g*best_^*t*−1^ in last generation:

(11) $$\begin{array}{c} Ld_{i}^{t+1}=\alpha Ld_{i}^{t},\\ R_{i}^{t+1}=\frac{1}{1+e^{\left(-(10/t_{\max})\times (t-(t_{\max}/2))+R_{i}^{1}\right)}}, \end{array}$$

where *α* ∈ [0,1] is a constant, *t* is optimizing generation, *t*_max⁡_ denotes maximum optimizing generation, and *R*_*i*_^1^ denotes initial pulse emission rate of each bat.

Cloud model bat algorithm is inspired by the behavior of bat; original BA and cloud model are used for reference, based on the properties of cloud model and echolocation of bat in foraging behavior, to remodel the algorithm framework, defining several idealized rules and constructing optimizing mechanism; a cloud model bat algorithm is proposed. The cloud model bat algorithm is different from the original bat algorithm, which uses echolocation predation mechanism of bats as the starting point and uses the universality of the normal cloud model as the basis. Several predominant mechanisms are integrated organically in the CBA.

For the performance of the proposed algorithm, the populations bats cloud *Pbc*_*j*_(*Ex*, *En*, *He*) utilizes the information provided by the current optimal solution to generate the drops of cloud. The loudness and pulse emission rate are regarded as entropy *En* and hyper entropy *He*, respectively, to control the measure of the coverage and randomness of optimal solution structure. Reduction of the loudness and increasing of the pulse rate emission show that bats approach their target. To quantificationally represent this qualitative concept by population, bats cloud model makes many individual clusters around the current optimal solution and forms a bat cloud, thus exploring much better solutions. This proposed algorithm has strong stability with bat cloud updating, which can gradually approach the optimal solution.

The swarm information communication guides the whole population moving toward the optimal solution. The increase or decrease of frequency *f* controls the scale of the individual moving forward or backward. Each individual can communicate information with others and ultimately move toward the common goal or direction. On the one hand, (7) implements on the basis of population elite *x*_*i*_^*t*^, which can accelerate the convergence speed of proposed algorithm; however, it may lead to premature convergence. On the other hand, the mechanism also reflects the importance of Lévy flight.

From (7), we know that premature convergence may take place; from (8), Lévy flight is implemented on the individual experience of population. This randomness of Lévy flight can ensure the diversity of the population against premature convergence. Lévy flight has a certain role in escaping from local optima; meanwhile, based on individual experience of population it can accelerate the convergence rate to some extent.

The range of loudness *Ld*_*i*_ and pulse emission rate *R*_*i*_ may have influence on the performance of the proposed algorithm; meanwhile, the number of the drops of cloud is no exception. In [13], for the drops of cloud are generated by the normal cloud generator, there are 99.7% located in the interval [*Ex* − 3*En*, *Ex* + 3*En*]. Consequently, initial value of *Ld*_*i*_ initializes 0.5(*x*_max⁡_ − *x*_min⁡_)/3, where *x*_max⁡_ and *x*_min⁡_ denote the upper and lower limit of the search space. The range of the drops of cloud that are located around the expectation *Ex* will reduce while the loudness *Ld*_*i*_ reduces gradually. In addition, the thickness of cloud cluster will increase while the pulse emission rate increases gradually; even the cloud cluster is excessively discrete and represents atomized feature. In [25], the expectation *Ex* can be approximated by reverse clouds generator and the error is less than 0.01 if only the number of the drops of cloud is more than 10. Similarly, to approximate entropy *En* and assure relative error less than 0.01, the number of the drops of cloud is more than 100. Consequently, this paper produces 100 drops of cloud in the implementation, as a cloud cluster, and randomly selects a larger sample with 50 drops of cloud from the cloud cluster to fit the structure individual.

## 4. Simulations and Result Analysis

In order to validate the validity of bat cloud model algorithm, several unconstrained high-dimensional benchmark test functions are selected (simulation platform: Microsoft Windows XP Professional SP3, AMD Athlon (tm) II X4 640 3.00 GHz, 4.00 GB; programming tools: Matlab R2012a).

### 4.1. Parameter Settings and Analysis

In this section, in order to test the sensibility of parameter settings, the 2-dimensional Rosenbrock function was selected. And its global minimum value is 0 at (1,1,…, 1); the global minimum is inside a long, narrow, and parabolic shaped flat valley. To find the valley is trivial. To converge to the global minimum, however, is difficult. Statistical result of minimum fitness after 50 independent runs was represented by box-and-whisker diagram, and the iterative curve was depicted for once independent run.

**Figure 3:**
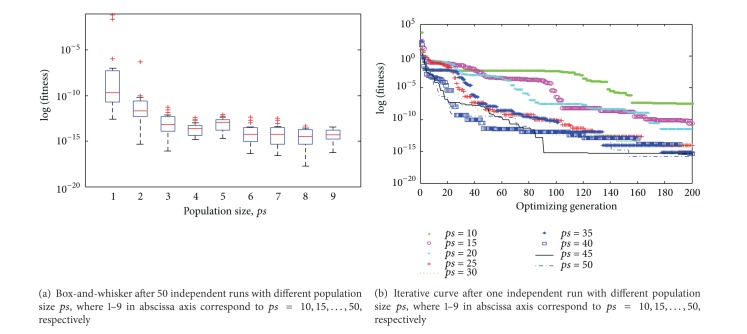
Box-and-whisker diagram and iterative curve about the impact of different population size  *ps*.

To initialize the parameter frequency *f* ∈ [0,2], *nt* = 4000, select different population size *ps* for experiment. The purpose is to investigate the influence of the population size for the proposed algorithm. The descriptive statistics of the results are plotted in Figure 3(a), where 1–9 in abscissa axis correspond to  *ps* = 10,15,…, 50, respectively. As shown as Figure 3(a), the precision of the optimum value gradually increases while the population size  *ps*  increases, and the increment of precision gradually decreases. The precision of the optimum value is low and the extreme outliers will appear when population only includes 10 individuals, which show that the population size is insufficient and the exploring ability is poor. The precision of the optimal value increases properly and the outliers are mild when population size increases to 20. After the population size reaches 30, performance of the proposed algorithm gradually stabilizes, and the incremental extent of the precision is inapparent.

Figure 3(b) is the iterative curves for one independent run of CBA. As shown in the figure, the difference of the optimum value is not outstanding after the population size increases to 30. Considering the precision and calculation, *ps* = 45 is a preferable balance, which has high precision and less calculation; in addition, the convergence rate is relatively fast.

**Figure 4:**
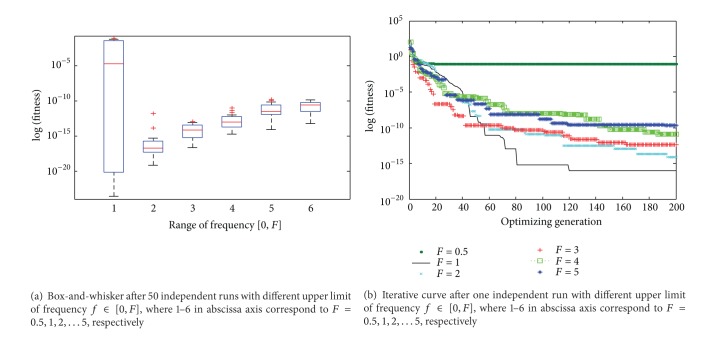
Box-and-whisker diagram and iterative curve about the impact of different upper limit of frequency *f* ∈ [0, *F*].

After setting the parameter *ps* = 45, *nt* = 4000 to test the proposed algorithm with different upper limit of frequency *f* ∈ [0, *F*]. The purpose is to investigate the impact of the frequency range for the proposed algorithm. The descriptive statistics of the results are plotted in Figure 4(a), where 1–6 in abscissa axis correspond to *F* = 0.5,1, 2,…, 5, respectively. As shown in Figure 4(a), the algorithm is very sensitive to the initial values, while *f* ∈ [0,0.5], the exploring ability is weak and cannot avoid the premature convergence and escape from local minima, and the stability is poor. The performance of algorithm is relatively good when the range is [0,1]; the performance of CBA gradually reduces while the upper limit of frequency increases.  Figure 4(b) is the iterative curves for one independent run of CBA.  Figure 4(b) shows that the performance of algorithm and convergence speed are preferable to the other condition.

**Figure 5:**
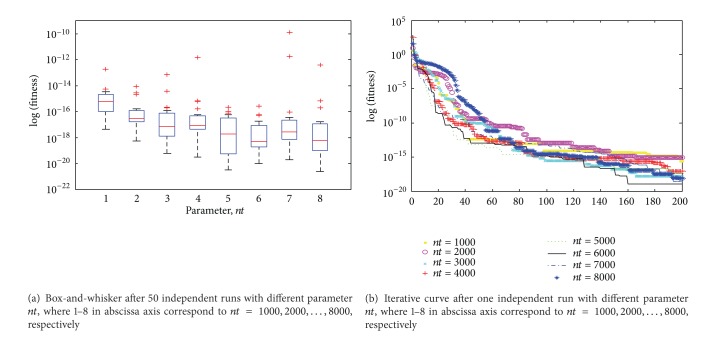
Box-and-whisker diagram and iterative curve about the impact of different parameter *nt*.

Confirm the parameter *ps* = 45, *f* ∈ [0,1], and then investigate the impact of the parameter *nt* for the proposed algorithm. The descriptive statistics of the results are plotted in Figure 5(a), where 1–8 in abscissa axis correspond to *nt* = 1000,2000,…, 8000, respectively.  Figure 5(b) is the iterative curves for one independent run of the proposed algorithm with different parameter *nt*. As shown in Figure 5, the performance of the algorithm has improved to some extent with gradually increasing parameter *nt*. In general, the algorithm parameter is not sensitive for parameter *nt*. Considering the precision of optimal value, convergence speed, and stability, parameter *nt* around 5000 or 6000 is preferable. In this paper, *nt* = 6000.

### 4.2. Combination Strategies Analysis

**Figure 6:**
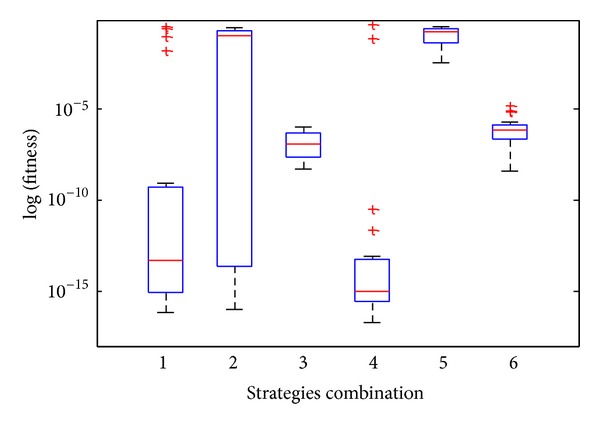
Box-and-whisker after 20 runs independently about different strategies combination.

In order to discuss the impact of three strategies (bats cloud updating, swarm information communication, and bats random Lévy flight) after selecting proper parameter, firstly, set bats cloud updating as** C**, swarm information communication as** G**, and bats random Lévy flight as** L** and then select Rosenbrock as test function. The statistical results after 20 times run independently are shown in Figure 6, where 1–6 in abscissa axis correspond to** LGC, CLG, CGL, GL, CL,** and** CG**, respectively, where the combination of letters represents the combination of different strategies. As shown as Figure 6,** LGC** sometimes can find a better solution, but it is unstable, and several extreme outliers appear sometimes, which represent that the algorithm may be premature convergence. The** CLG** is the most unstable, which is sensitive to the initial position.** GL** can repeatedly find a better solution; however, several extreme outliers appear likewise, which represent that the algorithm lacks stability.** CL** optimizes difficultly and its performance is poor. With** CG** several mild outliers will appear and the performance of** CG** is somewhat less than** CGL**, and the reason is lack of diversity without** L**. Nevertheless, the** CGL** loses trifling precision of the optimum value, but the overall performance is the most stable, and it can always find better solution.

### 4.3. Experimental Results and Analysis

In order to compare the performance with other algorithms, eight test functions are selected to test CBA convergence. In Table 1, the values listed in the search space column are used to specify the range of the initial random particles' position; the *x*_∗_ denotes the global optimum, and the *f*_min⁡_ is the corresponding fitness value.

In [11], a cloud model based genetic algorithm (CGA) was proposed; CGA is based on both the idea of GA and the properties of randomness and stable tendency of a normal cloud model. In [12], a cloud model based evolutionary algorithm (CBEA) was proposed by Zhang et al., which is based on the outstanding characteristics of the cloud model on the process of transforming a qualitative concept to a set of quantitative numerical values and integrates with the basic principle of evolutionary computation. In [13], cloud based evolutionary algorithm (CBEA) was proposed by Liu et al., who discuss the atomized feature of cloud model; the selection pressure of evolution is adjusted by changing the hyper entropy that is the main factor in atomized feature.

There are many ways to carry out the comparison of algorithm performance with different termination criteria; the preferable approaches is to compare their accuracies for a fixed number of fitness function evaluations *FEs*. This paper adopts fixed *FEs* as the termination criterion (*FEs* = *ps* × *t* × *N*, where *ps* is population size, *t* is optimizing generation, and *N* is the number of fitness function evaluations in each optimizing generation). In order to compare with different algorithms, search space (SS) and function dimension (FD) of selected benchmark functions are consistent with corresponding algorithm. CBA is run 50 times independently for each function and the mean of the function values found in 50 runs was described in experimental result.

In [11], *ps* was set as 100, selected different test functions have different optimizing generation *t*, the minimum *FEs* is 10^6^, and the maximum  *FEs*  is  2 × 10^7^. In this paper, optimizing generation *t* is set as 200 in each run for corresponding function. *FEs* = 27000. The experimental results are presented in Table 2, where results of CGA in 30 runs are derived from [11] and results of SAPSO (simulated annealing particle swarm optimization) are derived from [6].

Table 3 shows the results of comparison between CBEA08 and CBA, where results of CBEA08 in 50 runs are derived from [12], and results of SAPSO are derived from [6]. Here *ps* = 1000, *t* = 100, and *FEs* = 10^5^. In this paper, for these functions, *t* is set as 500, corresponding to *FEs* = 67500.

Table 4 shows the results of comparison between CBEA09 and CBA, where results of CBEA09 in 50 runs are derived from [13], and results of SAPSO are derived from [6]. All the selected functions are 30-dimensional function, and the optimizing generation  *t*  is set as 500 for all algorithms.

Tables 2, 3, and 4 not only show that the proposed algorithm is feasible and effective but also demonstrate the superior approximation capabilities in high-dimensional space. The proposed algorithm can perform better performance while the optimizing generation *t* increases gradually. As shown as Table 2, CBA has higher precision than CGA both unimodal and multimodal functions. From Table 3, CBA has similar precision than CBEA08 both unimodal and multimodal function, and several theoretical values of benchmark function can be achieved. Table 4 shows the results that are tested under high-dimensional condition. The CBA can perform with better precision, except for Rosenbrock function which has no best performance. Rosenbrock function has a narrow valley from the perceived local optima to the global optimum.

## 5. Conclusions

In this paper, the bat algorithm is used for reference; two mechanisms were introduced, population information communicating of each individual and random Lévy flights, to propose a cloud model bat algorithm based on normal cloud model and echolocation mechanism. Cloud model builds a transformational bridge between a linguistic term of qualitative concept and quantitative representation, which reflects randomness, fuzziness, and the relationship between randomness and fuzziness of uncertainty in knowledge representation. A population bats cloud model *Pbc*_*j*_(*Ex*, *En*, *He*) was built, which analogizes to cloud *C*(*Ex*, *En*, *He*), which depicts the qualitative concept: “bats approach their prey.” CBA mainly considers the balance between the global random search and local search and the balance between intensification and diversification. In addition, we discuss the mechanism and parameter set of CBA; several appropriate parameters are set. The simulation results show that the proposed algorithm is feasible, effective, and robust.

## Figures and Tables

**Algorithm 1:**
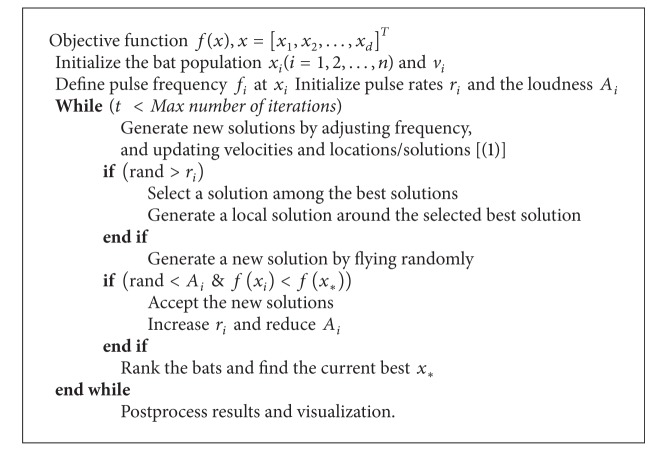
Pseudocode of the bat algorithm (BA) [3].

**Table 1:** Benchmarking test functions.

Benchmarks functions	Functions expression	Exact value	*x*_∗_	Search space
*f*_1_: Sphere	$f(x)=\overset{n}{\underset{i=1}{\sum }}x_{i}^{2}$	*f*_min⁡_ = 0	(0,0,…, 0)	[−10, 10]
*f*_2_: Schwefel	$f(x)=\overset{n}{\underset{i=1}{\sum }}\left|x_{i}\right|+\prod_{i=1}^{n}\left|x_{i}\right|$	*f*_min⁡_ = 0	(0,0,…, 0)	[−10, 10]
*f*_3_: Rosenbrock	$f(x)=\overset{n}{\underset{i=1}{\sum }}\left[{{(x}_{i}-1)}^{2}+100{\left(x_{i+1}-{x_{i}}^{2}\right)}^{2}\right]$	*f*_min⁡_ = 0	(1,1,…, 1)	[−2.408, 2.408]
*f*_4_: Ackley	f(x)=20+e-20exp⁡[-0.2(1n)×∑i=1nxi2]=-exp⁡[-0.2(1n)×∑i=1ncos⁡(2πxi)]	*f*_min⁡_ = 0	(0,0,…, 0)	[−30, 30]
*f*_5_: Griewangk	$f(x)=\frac{1}{4000}\times \overset{n}{\underset{i=1}{\sum }}x_{i}^{2}-\prod_{i=1}^{n}\cos\frac{x_{i}}{\sqrt{i}}+1$	*f*_min⁡_ = 0	(0,0,…, 0)	[−600, 600]
*f*_6_: Rastrigin	$f(x)=10n+\overset{n}{\underset{i=1}{\sum }}\left[x_{i}^{2}-10\cos\left(2\pi x_{i}\right)\right]$	*f*_min⁡_ = 0	(0,0,…, 0)	[−5.12, 5.12]
*f*_7_: Shubert	$f\left(x,y\right)=\left[\overset{5}{\underset{i=1}{\sum }}i\cos\left(i+\left(i+1\right)x\right)\right]\cdot \left[\overset{5}{\underset{i=1}{\sum }}i\cos\left(i+\left(i+1\right)y\right)\right]$	*f*_min⁡_ ≈ −186.7309	—	[−10, 10]
*f*_8_: Easom	*f*(*x*, *y*) = −cos⁡(*x*)cos⁡(*y*)exp⁡[−(*x*−*π*)^2^ + (*y*−*π*)^2^]	*f*_min⁡_ = −1	(*π*, *π*)	[−10, 10]

**Table 2:** Experimental results comparison between CGA, CBA, and SAPSO.

Algorithms	*f*_2_	*f*_3_	*f*_5_	*f*_6_	*f*_7_
S-S	[−10, 10]	[−2.048, 2.048]	[−512, 512]	[−10, 10]	[−10, 10]
F-D	5	2	10	15	2
CGA	0.00013	2.5749*e* − 08	0.01170	1.8529*e* − 06	−186.6267
SAPSO	—	3.5383*e* − 003	5.7773*e* − 008	1.9425*e* − 004	—
CBA	1.9850*e* − 93	1.2683*e* − 12	0	0	−186.7309

**Table 3:** Experimental results comparison between CBEA08, CBA, and SAPSO.

Algorithms	*f*_1_	*f*_5_	*f*_6_	*f*_7_	*f*_8_
S-S	[−100, 100]	[−600, 600]	[−5.12, 5.12]	[−10, 10]	[−100, 100]
F-D	10	10	10	2	2
CBEA08	0	0	0	−186.7309088310227	−1
SAPSO	0.04860173	5.7773*e* − 008	1.9425*e* − 004	—	—
CBA	0	0	0	−186.7309088310230	−1

**Table 4:** Experimental results comparison between CBEA09, CBA, and SAPSO.

Algorithms	*f*_1_	*f*_2_	*f*_3_	*f*_4_	*f*_5_
S-S	[−5.12, 5.12]	[−10, 10]	[−30, 30]	[−32.768, 32.768]	[−32.768, 32.768]
CBEA09	1.1696*e* − 166	5.2927*e* − 97	26.178	0	0
SAPSO	0.04860173	—	3.5383*e* − 003	1.5684*e* − 003	5.7773*e* − 008
CBA	0	2.4707*e* − 223	28.879	0	0
